# Effect of Artificial Liver Support Systems on Gut Microbiota in Patients with HBV-Related Acute-on-Chronic Liver Failure

**DOI:** 10.3390/pathogens12091094

**Published:** 2023-08-28

**Authors:** Zhiying Song, Qiong Xie, Yao Zhou, Shufen Song, Zhen Gao, Yu Lan, Zhiguo Wu, Hongxin Cai, Dongshan Yu, Cuiyun Liu, Junrong Liang, Baogang Xie, Shuilin Sun

**Affiliations:** 1 Department of Infectious Diseases, The Second Affiliated Hospital of Nanchang University, Nanchang 330006, China; songzhiying717@163.com (Z.S.); 13970062103@163.com (Q.X.); zy19991999zy@163.com (Y.Z.); lanyu7160@163.com (Y.L.); 18270632702@163.com (Z.W.); jdyudongshan@hotmail.com (D.Y.); liucuiyun2008-172@163.com (C.L.); 2 School of Pharmaceutical Sciences, Zhejiang Chinese Medical University, Hangzhou 311400, China; 15258374598@163.com; 3 State Key Laboratory for Infectious Disease Prevention and Control, National Institute for Communicable Disease Control and Prevention, Chinese Center for Disease Control and Prevention, Beijing 102206, China; 4 Department of Pharmaceutics, Medical College of Jiaxing University, Jiaxing 314033, China

**Keywords:** gut microbiota, hepatitis B virus, acute-on-chronic liver failure, artificial liver support system, blood biochemical indicators

## Abstract

Hepatitis B virus-related acute-on-chronic liver failure (HBV-ACLF) is a rare and severe form of end-stage liver disease with high mortality; gut microbes are strongly associated with the development of this severe liver disease but the exact association is unclear. Artificial liver support systems (ALSS) are clinically important in prolonging the waiting time for liver transplantation and in aiding drug therapy to achieve remission. The aim of this study was to investigate the effect of ALSS on the abundance and diversity of microorganisms in the gut of HBV-ACLF patients. In this study, 109 stool samples were collected from patients with hepatitis B virus-associated acute chronic liver failure (HBV-ACLF) for 16S rRNA sequencing. Among them, 44 samples were from patients treated with ALSS therapy as an adjunct to standard medical treatment (SMT) and 65 were from patients receiving SMT only. Analysis of the sequencing results suggested that there were significant differences in the abundance and diversity of gut microbiota between the with-ALSS and without-ALSS groups (*p* < 0.05). The operational taxonomic units and Shannon indexes indicated that the diversity and abundance of the gut microbiome, while decreasing after the first ALSS treatment, gradually increased after an increase in the number of ALSS therapies. The overall proportion of HBV-ACLF patients with coinfection was 27.59%; the coinfection can reduce the abundance of the *Bacteroidetes* phylum in the microbiome significantly whereas *Proteobacteria* were highly enriched. After ALSS therapy, HBV-ACLF patients had a decrease in potentially harmful bacteria, an increase in potentially beneficial bacteria, an increase in the diversity of the intestinal microbiota, and the intestinal microecological disorders were corrected to a certain extent. Serum alanine aminotransferase (ALT), aspartate aminotransferase (AST), and total bilirubin (TBIL) levels, as well as the international normalized ratio (INR), showed a decreasing trend whereas plasminogen activity (PTA) increased and the condition of patients with HBV-ACLF progressed in a favorable direction. In addition, the abundance of *Blautia* and *Coprococcus* was negatively correlated with TBIL and INR, positively correlated with PTA, and positively correlated with disease recovery. Our study shows that ALSS can alter the composition of the gut microbiota and have an ameliorating effect on the gut microecological imbalance in HBV-ACLF patients. It is worth mentioning that *Blautia* and *Coprococcus* may have great potential as biomarkers.

## 1. Introduction

Chronic hepatitis B virus (HBV) infection remains a serious global public health problem with a high prevalence, large number of infected people, and high mortality rate when the disease progresses to end-stage liver disease [1,2,3]. HBV-related acute-on-chronic liver failure (HBV-ACLF) is an end-stage liver disease occurring on the basis of chronic HBV infection, with extensive hepatocellular necrosis, severe jaundice, and coagulopathy (prothrombin activity (PTA) <40% or international normalized ratio (INR) ≥1.5) in a short period of time, accompanied by serious complications such as ascites and/or hepatic encephalopathy and hepatorenal syndrome [4]. HBV-ACLF progresses rapidly, with a 28-day mortality rate up to 15 times that of patients with other chronic liver diseases [5,6,7].

Patients with HBV-ACLF are generally poorly treated with medical drugs and liver transplantation is the only treatment option with good outcomes [7,8,9] but the dilemma with liver transplantation is that it is time-critical, scarce, and costly. Artificial liver support systems (ALSS) are an effective treatment for liver failure. They can be used to extend the waiting time for a liver transplant or as an adjunct to standard medical treatment (SMT) to buy time for remission and to avoid a liver transplant. A number of randomized controlled trials in liver failure have demonstrated that ALSS can lead to improvements in severe complications of liver failure such as hepatic encephalopathy, hepatorenal syndrome, circulatory dysfunction, and immune dysfunction [10,11,12,13,14].

Human gastrointestinal tract microorganisms number up to 10^14^ [15,16] and these gut microorganisms are involved in the regulation of a number of physiological functions of the organism [16,17]. In recent years, with the increasing use of 16S rRNA gene amplicon sequencing and metagenomic sequencing of human stool samples [18,19,20], the understanding of the composition of the intestinal microbiota has been greatly improved and it has been found that the richness and diversity of the human intestinal flora are influenced by various factors such as the environment [21], diet [22], disease [23,24], medication [25,26], and age [27]. Both 16S rRNA gene amplicon sequencing and metagenomic sequencing suggest that the gut microbiota plays a very important role in both the pathogenesis and progression of liver disease and that patients with liver failure have a significantly altered gut microbiota composition compared to patients with chronic hepatitis, cirrhosis, and healthy populations [28]. In end-stage liver disease, the use of gut microbiota composition to assess gut microecological stability, resilience, or health status and thus to guide the diagnosis and treatment of the disease and assess prognosis is very promising and has been the focus of research by experts in various countries in recent years [28,29,30,31].

Therefore, the aim of this study was to analyze the impact of ALSS on the gut microbiome of patients with HBV-ACLF by high-throughput 16S rRNA sequencing, and research the difference in gut microbial composition and blood biochemical indicators in the patients coinfected by ACLF and those without coinfection.

## 2. Materials and Methods

### 2.1. Patients and Samples Collection

From November 2017 to April 2018, 91 volunteers with HBV-ACLF were recruited by the Department of Infectious Diseases of the Second Affiliated Hospital of Nanchang University to leave stool samples and 109 samples left by 58 of these patients were randomly selected for the experiment. All volunteers with HBV-ACLF who retained stool samples were not taking antibiotic medication in the three weeks prior to sample retention. All HBV-ACLF volunteers met the consensus of the Asia Pacific Association for the Study of the Liver (APASL) [4]: the patient has been persistently positive for hepatitis B surface antigen (HBsAg) and/or HBV DNA for more than 6 months and severe jaundice (TBIL ≥ 5mg/dL) as well as coagulation dysfunction (PTA < 40% or INR ≥ 1.5) with ascites and/or hepatic encephalopathy within 4 weeks. All treatment measures taken comply with APASL recommendations. Coinfection is considered when patients with HBV-ACLF present with fever, increased respiratory rate or hyperventilation, elevated heart rate, changes in white blood cell (WBC), or increased infection indicators such as neutrophil percentage (NEUT%), calcitoninogen, c-reactive protein, endotoxin, etc.; blood, ascites, or sputum bacterial cultures and drug sensitivity tests can identify the infecting agent [4,32,33,34].

In addition to collecting stool samples, we also collected blood biochemical results from all patients, including alanine aminotransferase (ALT), aspartate aminotransferase (AST), albumin (ALB), TBIL, PTA, INR, WBC, NEUT%, platelets (PLT), and blood ammonia (BA) as well as information on the patients’ clinical profile, including clinical manifestations such as nausea, vomiting, poor appetite, and clinical prognosis. The patients with HBV-ACLF were considered to have a good prognosis by being discharged in remission and a poor prognosis by being discharged with uncontrolled disease or even death.

In this study, we tested several blood biochemical parameters at the time point of the patient’s stool sample collection, including ALT and AST, which are related to the inflammatory response of the liver; ALB, which is related to the antioxidant effect of the liver and the nutritional status of the body; TBIL, PTA, and INR, which are related to the severity of liver failure; and WBC and NEUT%, which are related to infection. We investigated the correlation between these blood biochemical parameters and the abundance of intestinal flora.

### 2.2. 16S rRNA Sequencing

To analyze the gut microbiota, 109 stool samples left from HBV-ACLF patients were subjected to 16S rRNA sequencing. Total bacterial genomic DNA was extracted from fecal samples using a fecal microbial genomic DNA extraction kit (QIAamp DNA Stool MiniKit). DNA samples were quantified using a Qubit 2.0 Fluorometer (Invitrogen, Carlsbad, CA, USA). The V3 and V4 highly variable regions of 16S rRNA were used as targets for PCR amplicons to amplify the V3–V4 highly variable regions of the 16S rRNA gene using forward primer 341F and reverse primer 806R. PCR amplification was performed according to the method described by Kozich et al. [35]. The PCR products were then examined for size and specificity by agarose gel electrophoresis and purified. Finally, amplicons were sequenced using the Illumina MiSeq according to the manufacturer’s instructions (Illumina, San Diego, CA, USA) [36]. Sequencing was performed using a 2 × 250 paired-end structure, with image analysis and base calling performed using the MiSeq control software embedded in the MiSeq instrument.

### 2.3. Statistical Analysis

The diversity of the gut microbiota of HBV-ACLF patients was analyzed using alpha and beta diversity analysis. Alpha diversity indices were calculated using QIIME (version 1.9.1), including the Simpson, Shannon, chao1, and observed otus diversity index. Beta diversity analysis was based on weighted and unweighted UniFrac distances for Principal Coordinate Analysis (PCoA) to compare all samples. Wilcoxon, paired, and Kruskal rank-sum tests were used to compare whether there were significant differences in gut microbiota richness at the phylum and genus levels between different subgroups. Clinical indices were calculated and visualized using R software (version 4.0.2). PCoA was performed using the ade4 package (version 5.5) in R software. A Wilcoxon rank-sum test was performed using the wilcox.test function in R software and correlation analyses were tested using Spearman’s correlation with the psych package (Version 2.1.9) also in R software.

The data on clinical characteristics were statistically analyzed using SPSS 25.0 statistical software. For continuous variables, data were expressed as mean ± standard deviation (SD). Differences were assessed using analysis of variance and Tukey’s post-hoc test. Drawing with GraphPad Prism 8.0 software. A *p* value < 0.05 is a significant difference between groups. In tables and figures, * indicates *p* < 0.05.

## 3. Results

### 3.1. Samples Information

In this study, we collected 109 stool samples from 58 patients with HBV-ACLF, of which 32 patients received ALSS and 63 stool samples were collected at different times and 26 patients did not receive ALSS with 46 stool samples were collected.

According to whether or not ALSS was administered, the 109 stool samples were divided into with-ALSS and without-ALSS groups, with 44 samples in the former group and 65 samples in the latter. The T0, T1, T2, and T3 groups were divided according to the number of artificial liver treatments performed, with 65 samples in the T0 group (without ALSS), 20 samples in the T1 group (with once ALSS), 12 samples in the T2 group (with twice ALSS), and 12 samples in the T3 group (with third ALSS). There were 15 patients who had stool samples taken both before and after ALSS therapy. These samples were divided into pre- and post-groups based on when the samples were collected around the time point when the ALSS was administered. Among the 109 stool samples, 16 were taken from coinfected patients, while the other 93 samples taken from the uninfected group. The 16 stool samples were taken from 16 patients.

### 3.2. Clinical Characteristics

#### 3.2.1. Clinical Characteristics of the with-ALSS and without-ALSS Groups

Compared to the with-ALSS group, the liver inflammation indicators AST and ALT were higher in the without-ALSS group (*p* < 0.05; Table 1).

#### 3.2.2. Clinical Characteristics of the Pre- and Post-Groups

**Figure 1:**
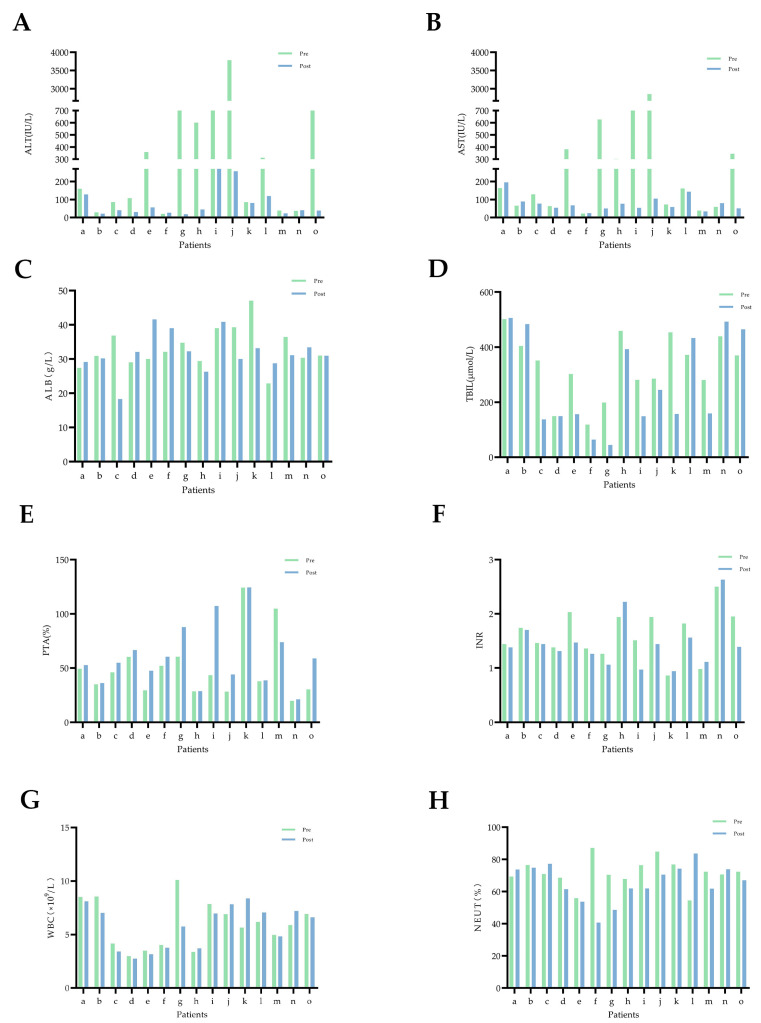
Changes in blood biochemical indicators in 15 patients with HBV-ACLF before and after artificial liver support therapy. Pre (before ALSS, green bar chart), post (after ALSS, blue bar chart), and the letters a–o represent each of these 15 HBV-ACLF patients. (**A**) ALT: alanine aminotransferase. (**B**) AST: aspartate aminotransferase. (**C**) ALB: albumin. (**D**) TBIL: total bilirubin. (**E**) PTA: prothrombin activity. (**F**) INR: international normalized ratio. (**G**) WBC: white blood cell. (**H**) NEUT: neutrophil.

The self-control experiment was conducted with 15 patients who collected stool samples both before and after artificial liver therapy. The clinical data of these patients showed that serum levels of ALT and AST were found to decrease in almost all the patients after ALSS therapy, TBIL levels and INR values decreased in the majority of patients, ALB and PTA increased, and WBC levels and NEUT% changed little (Figure 1).

#### 3.2.3. Clinical Characteristics of the T0, T1, T2, and T3 Groups

Compared to the T0 group, serum ALT levels were significantly lower in the T2 and T3 groups (*p* < 0.05; Table 2) and the NEUT% was significantly lower in the T1 group (*p* < 0.05; Table 2).

#### 3.2.4. Clinical Characteristics of the Infected and Uninfected Groups

Compared to the uninfected groups, TBIL levels and INR values were significantly higher in the infected group (*p* < 0.05; Table 3) while ALB levels, PTA, and PLT were higher in the uninfected group (*p* < 0.05; Table 3).

### 3.3. Distribution of HBV-ACLF Gut Microbiota at the Phylum and Genus Level

To investigate the differences in the abundance and diversity of gut microbiota at the phylum and genus levels among subgroups of HBV-ACLF patients, 109 stool samples were sequenced with 16S rRNA gene amplicons using alpha/beta diversity analysis and calculating the Simpson index, Shannon index, chao1 index, and otus index.

#### 3.3.1. Distribution of Gut Microbiota in the with-ALSS and without-ALSS Groups

**Figure 2:**
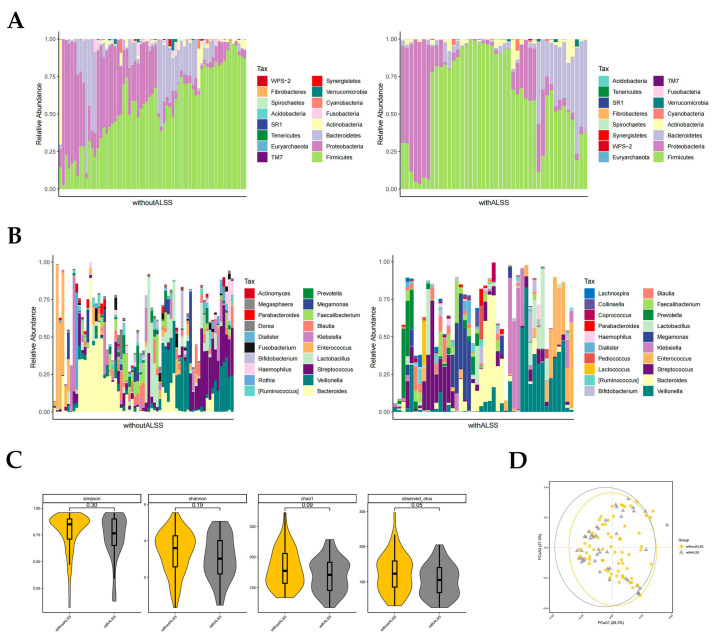
Distribution of gut microbiota in the with-ALSS and without-ALSS groups. Different colours in A and B represent different phyla or genera of gut microbes, and different colours in C and D represent different subgroups. (**A**) Relative abundance of gut microbiota at the phylum level in the with-ALSS and without-ALSS groups. (**B**) Relative abundance of gut microbiota at the genus level in the with-ALSS and without-ALSS groups. (**C**) Simpson, Shannon, chao1, and otus diversity index of the with-ALSS and without-ALSS groups. (**D**) PCoA plot based on the weighted unifrac distance of the with-ALSS and without-ALSS groups.

In the with-ALSS and without-ALSS groups, the top four highest gut microbiota phylum level richness were consistent, namely *Firmicutes*, *Proteobacteria*, *Bacteroidetes,* and *Actinobacteria* (Figure 2A). These two groups were significantly different in abundance with regard to *Proteobacteria* and *Fusobacteria* (*p* < 0.05, Wilcoxon rank-sum test; Figure 2A). Proteobacteria richness was higher in the without-ALSS group. *Fusobacteria* abundance was higher in the with-ALSS group. The top three most abundant HBV-ACLF fecal samples in terms of the genus level in both groups were *Bacteroides*, *Veillonella,* and *Streptococcus*. The results showed differences in the abundance of 11 genera including *Klebsiella*, *Blautia*, *Megasphaera*, *Serratia, Granulicatella,* etc., (*p* < 0.05, Wilcoxon rank-sum test; Figure 2B). In this study, *Enterococcus* was more abundant in the with-ALSS group and *Faecalibacterium* was more abundant in the without-ALSS group but neither was significantly different.

The gut microbiota diversity in the with-ALSS group versus the without-ALSS group showed a significant downward trend in the Simpson, Shannon, chao1, and otus diversity index (Figure 2C) and the PCoA plot also showed that patients in the with-ALSS group had lower gut flora diversity than the without-ALSS group (Figure 2D).

#### 3.3.2. Distribution of Gut Microbiota in the Pre- and Post-Groups

**Figure 3:**
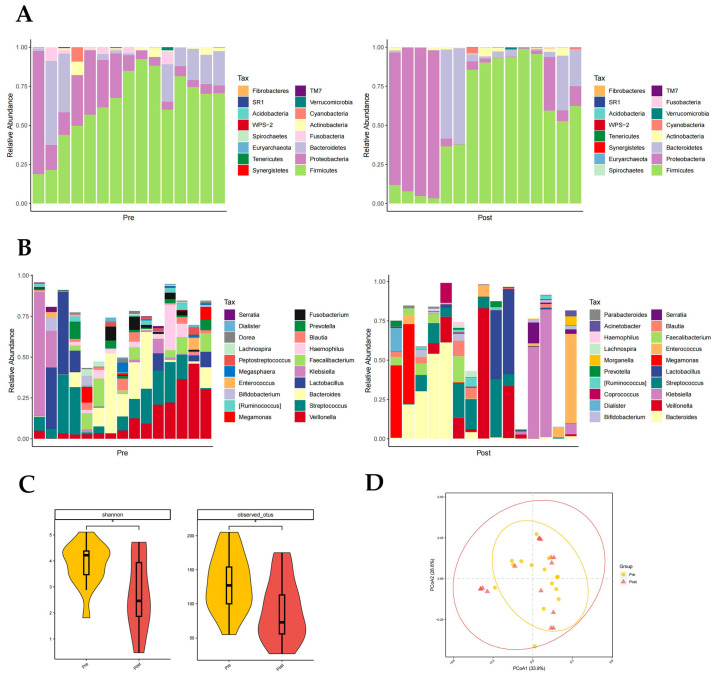
Distribution of gut microbiota in the pre- and post-groups. Different colours in A and B represent different phyla or genera of gut microbes, and different colours in C and D represent different subgroups. (**A**) Relative abundance of gut microbiota at the phylum level in the pre- and post-groups. (**B**) Relative abundance of gut microbiota at the genus level in the pre- and post-groups. (**C**) Shannon index (*p* = 0.037) and otus index (*p* = 0.028) of the pre- and post-groups (*p* < 0.05 from *t*-test). (**D**) PCoA plot based on the weighted unifrac distance of the pre- and post-groups.

*Firmicutes*, *Proteobacteria,* and *Bacteroidetes* had the highest phylum level richness in both the pre- and post-groups, with differences in *Fusobacteria* richness (*p* < 0.05, paired rank-sum test; Figure 3A). *Fusobacteria* are higher in the pre-group. There were significant differences in the abundance of 11 genera at the genus level, namely in *Rothia*, *Haemophilus*, *Megasphaera*, *Fusobacterium*, *Peptostreptococcus*, etc., (*p* < 0.05, paired rank-sum test; Figure 3B). It is worth noting that in the post group, the proportion of Enterococci was reduced. Both the Shannon index (*p* = 0.037) and otus index (*p* = 0.028) showed a significantly lower diversity of gut flora composition in the post group than in the pre-group (*p* < 0.05, Figure 3C). The PCoA plot also suggested that the post-group had a lower diversity of intestinal flora (Figure 3D).

#### 3.3.3. Distribution of Gut Microbiota in T0, T1, T2, and T3 Groups

**Figure 4:**
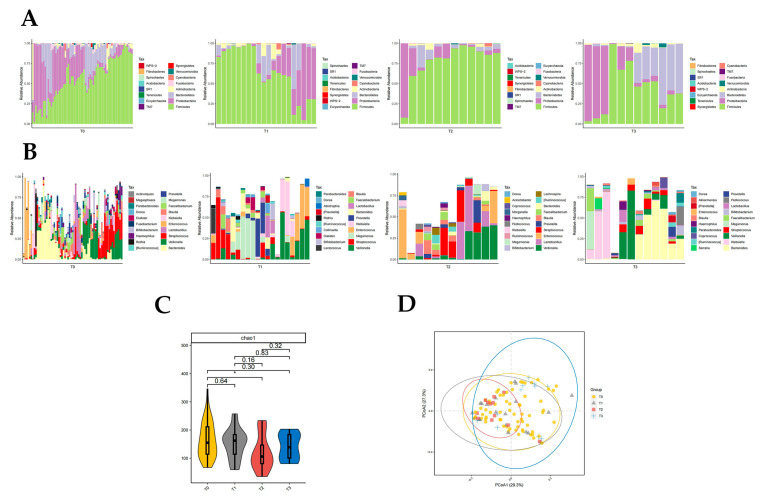
Distribution of gut microbiota in T0, T1, T2, and T3 groups. Different colours in A and B represent different phyla or genera of gut microbes, and different colours in C and D represent different subgroups. (**A**) Relative abundance of gut microbiota at the phylum level in T0, T1, T2, and T3 groups. (**B**) Relative abundance of gut microbiota at the genus level in T0, T1, T2, and T3 groups. (**C**) Chao1 index of T0, T1, T2, and T3 groups (*p* = 0.035 from *t*-test). (**D**) PCoA plot based on the weighted unifrac distance of T0, T1, T2, and T3 groups (*p* < 0.05, permutation test).

*Firmicutes* were significantly different in T0, T1, T2, and T3 groups (*p* < 0.05, Kruskal rank-sum test). The abundance of *Firmicutes* and *Proteobacteria* differed significantly between the T0 and T2 groups (*p* < 0.05, Wilcoxon rank-sum test; Figure 4A). *Fusobacteria* abundance differed significantly between T1 and T3 groups (*p* < 0.05, Wilcoxon rank-sum test; Figure 4A). The abundance of *Firmicutes* and *Fusobacteria* was significantly different in the T2 and T3 groups (*p* < 0.05, Wilcoxon rank-sum test; Figure 4A). There was no significant difference in intestinal flora richness at the phylum level between the T0 and T1 groups, T0 and T3 groups, and T1 and T2 groups compared to each other.

The abundance of 10 genera of *Klebsiella*, *Rothia*, *Megamonas*, *Morganella,* and *Granulicatella* was significantly different in groups T0, T1, T2, and T3 (*p* < 0.05, Kruskal rank-sum test). The abundance of *Megamonas*, *Leptotrichia*, *Slackia*, and *WAL_1855D* differed between the T0 and T1 groups (*p* < 0.05, Wilcoxon rank-sum test; Figure 4B). Seven genera of *Klebsiella*, *Lactococcus*, *Serratia*, *Enterobacter,* and *Elizabethkingia* differed in abundance between the T0 and T2 groups (*p* < 0.05, Wilcoxon rank-sum test; Figure 4B). There were differences in the abundance of 12 genera of *Rothia*, *Bifidobacterium*, *Blautia*, *Dialister,* and *Morganella* in the T0 and T3 groups (*p* < 0.05, Wilcoxon rank-sum test; Figure 4B). *Enterobacter* differed between the T1 and T2 groups, with Enterobacter being significantly less abundant after two artificial liver treatments compared to one artificial liver treatment (*p* < 0.05, Wilcoxon rank-sum test; Figure 4B). The nine genera of *Rothia*, *Megamonas*, *Bifidobacterium*, *Abiotrophia,* and *Fusobacterium* differed significantly in abundance between the T1 and T3 groups (*p* < 0.05, Wilcoxon rank-sum test; Figure 4B). There was a significant difference in *Rothia* and *Fusobacterium* abundance between the T2 and T3 groups (*p* < 0.05, Wilcoxon rank-sum test; Figure 4B).

The results of the chao1 diversity index analysis suggested that the T2 group had significantly lower gut microbial diversity than the T0 group (*p* = 0.035, Figure 4C). The PCoA plot also suggested that the T2 group was significantly less diverse than the T0 group (*p* = 0.048, Figure 4D). In addition, the PCoA plot also suggested a significant increase in gut microbial diversity in the T3 group compared to the T2 group (*p* = 0.02, Figure 4D), which was consistent with the trend in the results of the chao1 index analysis (Figure 4C).

#### 3.3.4. Distribution of Gut Microbiota in the Infected and Uninfected Groups

**Figure 5:**
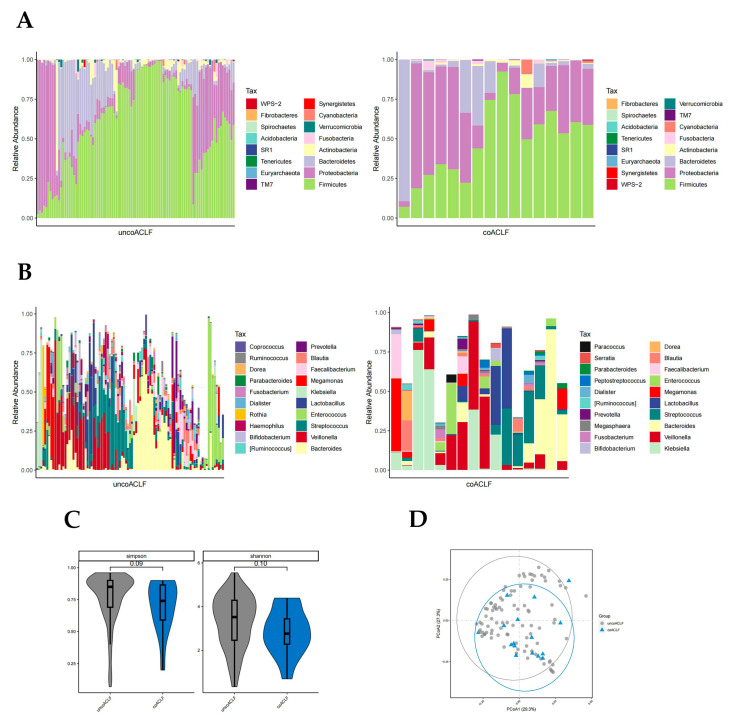
Distribution of gut microbiota in the infected and uninfected groups. Different colours in A and B represent different phyla or genera of gut microbes, and different colours in C and D represent different subgroups. (**A**) Relative abundance of gut microbiota at the phylum level in the infected and uninfected groups. (**B**) Relative abundance of gut microbiota at the genus level in the infected and uninfected groups. (**C**) Simpson and Shannon index of T0, T1, T2, and T3 groups. (**D**) PCoA plot based on the weighted unifrac distance of the infected and uninfected groups.

There was a significant difference in the abundance of *Bacteroidetes*, *Proteobacteria*, and *WPS-2* phylum in the coinfected and uninfected groups (*p* < 0.05, Wilcoxon rank-sum test; Figure 5A). The abundance of *Proteobacteria* was higher in the infected group than in the uninfected group. The abundance of *Bacteroidetes* was much higher in the uninfected group; *WPS-2* was low in both groups but slightly higher in the infected group. At the genus level, there were significant differences in the abundance of 28 bacteria genera (*p* < 0.05, Wilcoxon rank-sum test; Figure 5B) including *Ruminococcus*, *Megasphaera*, *Serratia*, *Coprococcus*, *Lachnospira*, etc., which were not highly enriched. The Simpson and Shannon diversity index and PCoA plot all suggest that coinfection leads to reduced intestinal flora diversity in patients with HBV-ACLF (Figure 5C,D).

### 3.4. Intestinal Flora Associated with Blood Biochemical Indicators

**Figure 6:**
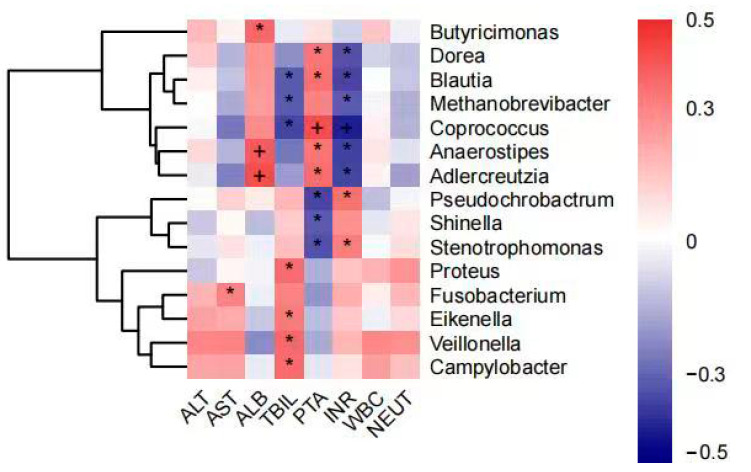
Correlation analysis of genus abundance with patient’s blood biochemical indicators (plus and asterisk both mean *p* < 0.05). * denotes statistical significance, + denotes more significant statistical significance.

The analysis showed that AST levels were positively correlated with the abundance of *Fusobacterium* and ALB levels were positively correlated with the abundance of *Butyricimonas*, *Anaerostipes,* and *Adlercreutzia*. TBIL levels were positively correlated with *Proteus*, *Eikenella*, *Veillonella*, and *Campylobacter* abundance and negatively correlated with *Blautia*, *Methanobrevibacter*, and *Coprococcus*. Coagulation dysfunction (PTA and INR) was negatively correlated with *Dorea*, *Blautia*, *Methanobrevibacter*, *Coprococcus*, *Anaerostipes*, and *Adlercreutzia* abundance and positively correlated with *Pseudochrobactrum*, *Shinella*, and *Stenotrophomonas*. In contrast, there was no significant correlation between ALT, WBC, and NEUT% or intestinal flora (Figure 6).

### 3.5. Clinical Outcome of Patients with HBV-ACLF

Only 1 of the 58 patients was treated with liver transplantation, 40 patients achieved remission with SMT or ALSS-assisted SMT, and 17 patients had a poor prognosis.

Of the 32 people with HBV-ACLF who received ALSS-assisted SMT, 1 patient completed liver transplantation, 20 had a good prognosis, and 11 had a poor prognosis. In total, 20 of the 26 patients who received SMT only had a good prognosis and 6 had a poor prognosis. In total, 9 of the 16 patients with coinfection had a poor prognosis and the patients who underwent liver transplantation were coinfected, so only 6 patients were in remission by SMT or ALSS-assisted SMT.

## 4. Discussion

The results of this study showed significant differences in the abundance and diversity of the gut microbiota between the with-ALSS and without-ALSS groups, the pre- and post-groups, the different numbers of artificial liver support treatments groups, and the infected and uninfected groups of HBV-ACLF patients (*p* < 0.05). In the with-ALSS and without-ALSS groups, there was a significant difference in the abundance of 2 phyla and 11 genera (*p* < 0.05), with the with-ALSS group having a lower intestinal flora diversity. In the pre- and post-groups, there was a significant difference in the abundance of 1 phylum and 11 genera (*p* < 0.05), with the post-group having significantly lower intestinal flora diversity than the pre-group (*p* < 0.05). In the T0, T1, T2, and T3 groups, 1 phylum and 10 genera richness were significantly different (*p* < 0.05). Gut microbial diversity was significantly lower in the T2 group than in the T0 group (*p* < 0.05), while it was significantly higher in the T3 group compared to the T2 group (*p* < 0.05). In total, 2 phyla and 28 genera differed significantly in abundance between the infected and uninfected groups (*p* < 0.05), with a decrease in intestinal flora diversity in the infected group compared to the uninfected group. It suggests that artificial liver support therapy is known to cause changes in the microbial composition of the gut and that such changes may contribute to remission in patients with HBV-ACLF.

However, due to the high cost of artificial liver support therapy, most of the HBV-ACLF patients in this study opted for artificial liver support therapy only after the ineffectiveness of SMT alone. Therefore, among these 58 HBV-ACLF patients, those in the with-ALSS group were more severely ill than those in the without-ALSS group (Table 1). And the only critically ill patient to receive a liver transplant was also a patient who received artificial liver support therapy. The poor clinical prognosis of patients in the with-ALSS group reached 34.375% compared to 23.077% in the without-ALSS group. The poor prognosis rate in the infected group was 56.25% while 13 of the 16 samples in the infected group were from the with-ACLF group. All this evidence suggests that intestinal flora translocation and intestinal microecological disorders were more severe in the with-ALSS group.

Based on the analysis of the 16S rRNA sequencing results, the phyla and genera with higher abundance in the intestinal microbiota of the with-ALSS and without-ALSS groups were the same while the phyla and genera with significant differences had a lower abundance (*p* < 0.05). Within the without-ALSS group, there is a much greater abundance of Proteobacteria, a highly variable phylum of bacteria containing many species of importance to human disease [37,38]. A higher abundance of Fusobacteria in the with-ALSS group was found to cause opportunistic infections and was significantly associated with colorectal cancer [39]. The lower intestinal flora diversity, higher *Enterococcus* abundance, and lower *Faecalibacterium* abundance in the with-ALSS group may be due to the greater severity of liver failure in this group.

It was concluded from the clinical data, from stool samples that were collected before and after artificial liver treatment in these 15 patients, that artificial liver support therapy was able to control liver severity, reduce the severity of liver failure, and improve coagulation. However, the diversity of the gut microbiota in the post group did not support this view, which we consider to be related to the delayed nature of changes in the gut microbiota compared to changes in blood biochemical parameters. The results of the paired rank-sum test analysis between the pre- and post-groups revealed a decrease in the enterococcal bacillus ratio in the post-group, suggesting that ALSS has a positive regulatory effect on enterococci and bacilli. Artificial liver support therapy will correct the unbalanced enterococci bacilli ratio in HBV-ACLF patients to some extent. Therefore, we conclude from the data of the pre- and post-groups that artificial liver support therapy can improve liver disease by altering the composition of the intestinal microbiota.

The effect of ALSS on the composition of the gut microbiota can be observed more clearly in the analysis of the results of the different numbers of artificial liver support treatment groups. In these four subgroups, there were significant differences in the abundance of the major gut phylum *Firmicutes*, *Proteobacteria*, and *Fusobacteria* (*p* < 0.05) and multiple genera with higher abundances differed between the two groups (*p* < 0.05), which is stronger evidence in favor of ALSS altering the composition of gut flora. In addition, the abundance of the potentially pathogenic bacterium *Enterobacter* decreased significantly after two artificial liver treatments compared to one artificial liver treatment (*p* < 0.05), intestinal flora diversity increased significantly after three artificial liver support treatments (*p* < 0.05), and liver inflammation were significantly controlled after three artificial liver treatments (*p* < 0.05), which supports that ALSS not only changes the intestinal microbial composition but also alters it in a direction that is beneficial for remission.

Coinfection in patients with HBV-ACLF is generally a sign of exacerbation (Table 3) and is considered to be associated with a large translocation of intestinal bacteria and bacterial derivatives [30,40]. Coinfected patients with HBV-ACLF had an. altered abundance of multiple bacterial genera (*p* < 0.05), increased potentially harmful bacteria, and reduced intestinal flora diversity, all suggesting a more severe condition in this group of patients. Artificial liver support therapy can reduce the infection to some extent (*p* < 0.05; Table 2).

ALSS causes changes in the composition of the intestinal flora but there are few species of flora with significant differences in comparative abundance between subgroups and the abundance of these flora is generally low. Artificial liver support therapy affects the composition of the intestinal flora but the ability to do so is limited. The ability of ALSS to modulate intestinal flora is somewhat consistent with the results of several large randomized trials on the efficacy of ALSS [12,13]. The results of several trials suggest that compared to liver transplantation and SMT, ALSS is not effective in improving the short-term survival of HBV-ACLF patients but it can significantly reduce serum bilirubin levels, improve clinical symptoms of complications such as hepatic encephalopathy and hepatorenal syndrome, and complement liver transplantation and drug therapy. The results of this study also support the recommendation that in clinical practice, patients with HBV-ACLF should be treated with ALSS in a timely manner if liver transplantation cannot be performed in a timely manner or if SMT therapy is ineffective.

When analyzing the correlation between blood biochemical parameters and intestinal microflora, it was found that the abundance of *Blautia*, *Coprococcus,* and *Methanobrevibacter* was negatively correlated with coagulation dysfunction and jaundice in HBV-ACLF patients, which also implies that the abundance of these bacteria was negatively correlated with the severity of liver failure. *Blautia*, *Coprococcus,* and *Methanobrevibacter* were reported as beneficial bacteria for improving the condition of HBV-ACLF. Studies have shown that *Blautia*, an anaerobic bacterium with probiotic properties, is able to modulate the immune system and alleviate inflammatory responses in the gut [41,42] and that *Coprococcus*, which has similar functions to *Faecalibacterium* [43], is one of the important producers of butyrate, with effects such as suppressing immune responses, reducing the severity of allergic reactions, and being antidepressant. These bacteria may be identified as biomarkers of remission in liver failure for disease assessment and can also be considered as therapeutic targets for the treatment of intestinal micro-ecological disorders.

In further studies, we will consider collecting fecal samples from healthy people and patients with chronic hepatitis and cirrhosis as well as fecal samples from patients with liver failure before and after treatment with ALSS to perform gene sequencing of the intestinal microbiota and comparatively analyze them to find genera that can be used as biomarkers. Subsequently, liver failure animal studies will be designed in anticipation of further confirming the importance of this intestinal bacterial genus in the development of liver failure disease. This research has the potential to lead to new clinical treatments.

In conclusion, artificial liver support systems are known to alter the composition of the gut flora in patients with HBV-ACLF and the modulation of the gut flora by ALSS can help improve liver failure. In addition, we found that the abundance of *Blautia*, *Coprococcus,* and *Methanobrevibacter* was negatively correlated with the severity of liver failure. These genera have the potential to be markers of good prognosis, markers for assessing disease, and therapeutic targets. Currently, there are limitations in that fewer patients were included in this study, even fewer had stool samples taken before and after ALSS treatment, and samples from different stages of liver disease were not available. In subsequent studies, we will seek new clinical treatments by breaking through these limitations and finding suitable intestinal genera as biomarkers for intervention.

## Figures and Tables

**Table 1:** Clinical characteristics of HBV-ACLF patients in the with and without ALSS groups.

Variables	With-ALSS	Without-ALSS	*p*-Value
Age (year)	43.84 ± 10.39	48.59 ± 13.45	
Male (Number)	41 (93%)	51 (78%)	
ALT (IU/L)	97.02 ± 113.15	444.68 ± 922.20	0.010 *
AST (IU/L)	93.96 ± 113.36	313.51 ± 693.95	0.030 *
ALB (g/L)	32.67 ± 4.50	33.75 ± 4.59	0.267
TBIL (μmol/L)	259.11 ± 174.39	226.28 ± 128.20	0.306
PTA (%)	65.95 ± 38.50	71.55 ± 38.42	0.483
INR	1.46 ± 0.51	1.35 ± 0.41	0.257
WBC (×10^9^/L)	5.40 ± 2.09	5.97 ± 2.11	0.195
NEUT (%)	64.47 ± 10.99	68.01 ± 8.48	0.079
PLT (×10^9^/L)	127.82 ± 84.60	161.53 ± 86.69	0.059
BA (μmol/L)	69.24 ± 29.57	62.85 ± 26.56	0.460

* The difference is statistically significant among the groups with ANOVA. ALT: alanine aminotransferase; AST: aspartate aminotransferase; ALB: albumin; TBIL: total bilirubin; PTA: prothrombin activity; INR: international normalized ratio; WBC: white blood cell; NEUT: neutrophil; PLT: platelet; BA: blood ammonia.

**Table 2:** Clinical characteristics of HBV-ACLF patients in the T0, T1, T2, and T3 groups.

Variables	T0	T1	T2	T3	*p*-Value
Age (year)	48.59 ± 13.45	44.85 ± 9.70	43.42 ± 13.59	42.58 ± 8.43	
Male (Number)	51 (78%)	17 (85%)	12 (100%)	12 (100%)	
ALT (IU/L)	444.68 ± 922.20	124.08 ± 140.52	80.90 ± 76.37 *	68.05 ± 87.04 *	0.036
AST (IU/L)	313.51 ± 693.95	121.20 ± 160.23	72.49 ± 42.28	70.05 ± 40.29	0.068
ALB (g/L)	33.75 ± 4.59	33.15 ± 3.79	31.92 ± 5.15	32.61 ± 5.17	0.605
TBIL (μmol/L)	226.28 ± 128.20	249.51 ± 193.58	238.11 ± 154.95	296.11 ± 167.37	0.551
PTA (%)	71.55 ± 38.42	61.89 ± 35.42	73.03 ± 31.10	65.63 ± 50.74	0.775
INR	1.35 ± 0.41	1.54 ± 0.56	1.32 ± 0.47	1.48 ± 0.46	0.379
WBC (×10^9^/L)	5.97 ± 2.11	5.09 ± 1.68	5.60 ± 1.71	5.72 ± 2.99	0.484
NEUT (%)	68.01 ± 8.48	60.91 ± 10.57 *	67.77 ± 10.58	67.10 ± 11.17	0.045
PLT (×10^9^/L)	161.53 ± 86.69	122.5 ± 91.99	150.33 ± 73.37	114.17 ± 84.66	0.193
BA (μmol/L)	62.85 ± 26.56	61.00 ± 23.75	83.33 ± 52.54	75.60 ± 25.21	0.508

* The differences between the marker and T0 groups were statistically significant with ANOVA. ALT: alanine aminotransferase; AST: aspartate aminotransferase; ALB: albumin; TBIL: total bilirubin; PTA: prothrombin activity; INR: international normalized ratio; WBC: white blood cell; NEUT: neutrophil; PLT: platelet; BA: blood ammonia.

**Table 3:** Clinical characteristics of HBV-ACLF patients in the infected and uninfected groups.

Variables	Coinfected	Uninfected	*p*-Value
Age (year)	50.00 ± 11.96	45.82 ± 13.57	
Male (Number)	13 (81%)	79 (84.9%)	
ALT (IU/L)	514.18 ± 1197.1	216.07 ± 507.85	0.364
AST (IU/L)	436.39 ± 974.37	159.80 ± 341.44	0.291
ALB (g/L)	31.06 ± 4.96	33.38 ± 4.71	0.035 *
TBIL (μmol/L)	317.11 ± 134.89	213.94 ± 148.93	0.003 *
PTA (%)	37.36 ± 25.99	75.61 ± 38.97	0.0001 *
INR	1.96 ± 0.48	1.3 ± 0.38	0.0001 *
WBC (×10^9^/L)	5.36 ± 2.38	5.68 ± 2.12	0.704
NEUT (%)	65.74 ± 9.95	64.98 ± 11.4	0.652
PLT (×10^9^/L)	92.56 ± 64.46	151.23 ± 87.63	0.012 *
BA (μmol/L)	65.90 ± 29.58	65.61 ± 27.60	0.864

* The difference is statistically significant among the groups with ANOVA. ALT: alanine aminotransferase; AST: aspartate aminotransferase; ALB: albumin; TBIL: total bilirubin; PTA: prothrombin activity; INR: international normalized ratio; WBC: white blood cell; NEUT: neutrophil; PLT: platelet; BA: blood ammonia.

## Data Availability

The raw 16S rRNA gene data reported in this paper were deposited in the Genome Sequence Archive in National Genomics Data Center for Bioinformation/Beijing Institute of Genomics, Chinese Academy of Sciences, under accession number CRA003748, which is publicly accessible at https://bigd.big.ac.cn/gsa (accessed on 24 August 2023).

## References

[1] (2017). EASL 2017 Clinical Practice Guidelines on the management of hepatitis B virus infection. J. Hepatol..

[2] Sarin S.K., Kumar M., Lau G.K., Abbas Z., Chan H.L., Chen C.J., Chen D.S., Chen H.L., Chen P.J., Chien R.N. (2016). Asian-Pacific clinical practice guidelines on the management of hepatitis B: A 2015 update. Hepatol. Int..

[3] Schweitzer A., Horn J., Mikolajczyk R.T., Krause G., Ott J.J. (2015). Estimations of worldwide prevalence of chronic hepatitis B virus infection: A systematic review of data published between 1965 and 2013. Lancet.

[4] Sarin S.K., Choudhury A., Sharma M.K., Maiwall R., Al Mahtab M., Rahman S., Saigal S., Saraf N., Soin A.S., Devarbhavi H. (2019). Acute-on-chronic liver failure: Consensus recommendations of the Asian Pacific association for the study of the liver (APASL): An update. Hepatol. Int..

[5] Wlodzimirow K.A., Eslami S., Abu-Hanna A., Nieuwoudt M., Chamuleau R.A. (2012). Systematic review: Acute liver failure—one disease, more than 40 definitions. Aliment. Pharmacol. Ther..

[6] Moreau R., Jalan R., Gines P., Pavesi M., Angeli P., Cordoba J., Durand F., Gustot T., Saliba F., Domenicali M. (2013). Acute-on-chronic liver failure is a distinct syndrome that develops in patients with acute decompensation of cirrhosis. Gastroenterology.

[7] Arroyo V., Moreau R., Jalan R. (2020). Acute-on-Chronic Liver Failure. N. Engl. J. Med..

[8] Artru F., Louvet A., Ruiz I., Levesque E., Labreuche J., Ursic-Bedoya J., Lassailly G., Dharancy S., Boleslawski E., Lebuffe G. (2017). Liver transplantation in the most severely ill cirrhotic patients: A multicenter study in acute-on-chronic liver failure grade 3. J. Hepatol..

[9] Reddy M.S., Rajalingam R., Rela M. (2015). Liver transplantation in acute-on-chronic liver failure: Lessons learnt from acute liver failure setting. Hepatol. Int..

[10] Flamm S.L., Yang Y.X., Singh S., Falck-Ytter Y.T. (2017). American Gastroenterological Association Institute Guidelines for the Diagnosis and Management of Acute Liver Failure. Gastroenterology.

[11] Hassanein T.I., Tofteng F., Brown R.S., McGuire B., Lynch P., Mehta R., Larsen F.S., Gornbein J., Stange J., Blei A.T. (2007). Randomized controlled study of extracorporeal albumin dialysis for hepatic encephalopathy in advanced cirrhosis. Hepatology.

[12] Bañares R., Nevens F., Larsen F.S., Jalan R., Albillos A., Dollinger M., Saliba F., Sauerbruch T., Klammt S., Ockenga J. (2013). Extracorporeal albumin dialysis with the molecular adsorbent recirculating system in acute-on-chronic liver failure: The RELIEF trial. Hepatology.

[13] Kribben A., Gerken G., Haag S., Herget-Rosenthal S., Treichel U., Betz C., Sarrazin C., Hoste E., Van Vlierberghe H., Escorsell A. (2012). Effects of fractionated plasma separation and adsorption on survival in patients with acute-on-chronic liver failure. Gastroenterology.

[14] Mitzner S.R., Stange J., Klammt S., Risler T., Erley C.M., Bader B.D., Berger E.D., Lauchart W., Peszynski P., Freytag J. (2000). Improvement of hepatorenal syndrome with extracorporeal albumin dialysis MARS: Results of a prospective, randomized, controlled clinical trial. Liver Transplant..

[15] Savage D.C. (1977). Microbial ecology of the gastrointestinal tract. Annu. Rev. Microbiol..

[16] Bäckhed F., Ley R.E., Sonnenburg J.L., Peterson D.A., Gordon J.I. (2005). Host-bacterial mutualism in the human intestine. Science.

[17] Neish A.S. (2009). Microbes in gastrointestinal health and disease. Gastroenterology.

[18] Poretsky R., Rodriguez R.L., Luo C., Tsementzi D., Konstantinidis K.T. (2014). Strengths and limitations of 16S rRNA gene amplicon sequencing in revealing temporal microbial community dynamics. PLoS ONE.

[19] Lepage P., Leclerc M.C., Joossens M., Mondot S., Blottière H.M., Raes J., Ehrlich D., Doré J. (2013). A metagenomic insight into our gut’s microbiome. Gut.

[20] Laudadio I., Fulci V., Palone F., Stronati L., Cucchiara S., Carissimi C. (2018). Quantitative Assessment of Shotgun Metagenomics and 16S rDNA Amplicon Sequencing in the Study of Human Gut Microbiome. Omics A J. Integr. Biol..

[21] Tyakht A.V., Kostryukova E.S., Popenko A.S., Belenikin M.S., Pavlenko A.V., Larin A.K., Karpova I.Y., Selezneva O.V., Semashko T.A., Ospanova E.A. (2013). Human gut microbiota community structures in urban and rural populations in Russia. Nat. Commun..

[22] David L.A., Maurice C.F., Carmody R.N., Gootenberg D.B., Button J.E., Wolfe B.E., Ling A.V., Devlin A.S., Varma Y., Fischbach M.A. (2014). Diet rapidly and reproducibly alters the human gut microbiome. Nature.

[23] Henao-Mejia J., Elinav E., Jin C., Hao L., Mehal W.Z., Strowig T., Thaiss C.A., Kau A.L., Eisenbarth S.C., Jurczak M.J. (2012). Inflammasome-mediated dysbiosis regulates progression of NAFLD and obesity. Nature.

[24] Abrahamsson T.R., Jakobsson H.E., Andersson A.F., Björkstén B., Engstrand L., Jenmalm M.C. (2014). Low gut microbiota diversity in early infancy precedes asthma at school age. Clin. Exp. Allergy J. Br. Soc. Allergy Clin. Immunol..

[25] Jernberg C., Löfmark S., Edlund C., Jansson J.K. (2007). Long-term ecological impacts of antibiotic administration on the human intestinal microbiota. ISME J..

[26] Jakobsson H.E., Jernberg C., Andersson A.F., Sjölund-Karlsson M., Jansson J.K., Engstrand L. (2010). Short-term antibiotic treatment has differing long-term impacts on the human throat and gut microbiome. PLoS ONE.

[27] Claesson M.J., Cusack S., O’Sullivan O., Greene-Diniz R., de Weerd H., Flannery E., Marchesi J.R., Falush D., Dinan T., Fitzgerald G. (2011). Composition, variability, and temporal stability of the intestinal microbiota of the elderly. Proc. Natl. Acad. Sci. USA.

[28] Wang K., Zhang Z., Mo Z.S., Yang X.H., Lin B.L., Peng L., Xu Y., Lei C.Y., Zhuang X.D., Lu L. (2021). Gut microbiota as prognosis markers for patients with HBV-related acute-on-chronic liver failure. Gut Microbes.

[29] Pascal V., Pozuelo M., Borruel N., Casellas F., Campos D., Santiago A., Martinez X., Varela E., Sarrabayrouse G., Machiels K. (2017). A microbial signature for Crohn’s disease. Gut.

[30] Trebicka J., Bork P., Krag A., Arumugam M. (2021). Utilizing the gut microbiome in decompensated cirrhosis and acute-on-chronic liver failure. Nat. Reviews. Gastroenterol. Hepatol..

[31] Solé C., Guilly S., Da Silva K., Llopis M., Le-Chatelier E., Huelin P., Carol M., Moreira R., Fabrellas N., De Prada G. (2021). Alterations in Gut Microbiome in Cirrhosis as Assessed by Quantitative Metagenomics: Relationship With Acute-on-Chronic Liver Failure and Prognosis. Gastroenterology.

[32] Jha A.K., Nijhawan S., Rai R.R., Nepalia S., Jain P., Suchismita A. (2013). Etiology, clinical profile, and inhospital mortality of acute-on-chronic liver failure: A prospective study. Indian J. Gastroenterol. Off. J. Indian Soc. Gastroenterol..

[33] Silvestre J.P., Coelho L.M., Póvoa P.M. (2010). Impact of fulminant hepatic failure in C-reactive protein?. J. Crit. Care.

[34] Fernández J., Prado V., Trebicka J., Amoros A., Gustot T., Wiest R., Deulofeu C., Garcia E., Acevedo J., Fuhrmann V. (2019). Multidrug-resistant bacterial infections in patients with decompensated cirrhosis and with acute-on-chronic liver failure in Europe. J. Hepatol..

[35] Kozich J.J., Westcott S.L., Baxter N.T., Highlander S.K., Schloss P.D. (2013). Development of a dual-index sequencing strategy and curation pipeline for analyzing amplicon sequence data on the MiSeq Illumina sequencing platform. Appl. Environ. Microbiol..

[36] Klindworth A., Pruesse E., Schweer T., Peplies J., Quast C., Horn M., Glöckner F.O. (2013). Evaluation of general 16S ribosomal RNA gene PCR primers for classical and next-generation sequencing-based diversity studies. Nucleic Acids Res..

[37] Hugon P., Dufour J.C., Colson P., Fournier P.E., Sallah K., Raoult D. (2015). A comprehensive repertoire of prokaryotic species identified in human beings. Lancet. Infect. Dis..

[38] Consortium H.M.P. (2012). Structure, function and diversity of the healthy human microbiome. Nature.

[39] Brennan C.A., Garrett W.S. (2019). Fusobacterium nucleatum—symbiont, opportunist and oncobacterium. Nat. Reviews. Microbiol..

[40] Tranah T.H., Edwards L.A., Schnabl B., Shawcross D.L. (2021). Targeting the gut-liver-immune axis to treat cirrhosis. Gut.

[41] Bolte L.A., Vich Vila A., Imhann F., Collij V., Gacesa R., Peters V., Wijmenga C., Kurilshikov A., Campmans-Kuijpers M.J.E., Fu J. (2021). Long-term dietary patterns are associated with pro-inflammatory and anti-inflammatory features of the gut microbiome. Gut.

[42] Liu X., Mao B., Gu J., Wu J., Cui S., Wang G., Zhao J., Zhang H., Chen W. (2021). Blautia-a new functional genus with potential probiotic properties?. Gut Microbes.

[43] Valles-Colomer M., Falony G., Darzi Y., Tigchelaar E.F., Wang J., Tito R.Y., Schiweck C., Kurilshikov A., Joossens M., Wijmenga C. (2019). The neuroactive potential of the human gut microbiota in quality of life and depression. Nat. Microbiol..

